# Low serum calcium is associated with left ventricular systolic dysfunction in a Chinese population with coronary artery disease

**DOI:** 10.1038/srep22283

**Published:** 2016-02-29

**Authors:** Yong Wang, Heng Ma, Xiaochen Hao, Jun Yang, Qiujing Chen, Lin Lu, Ruiyan Zhang

**Affiliations:** 1 Department of Cardiology, Rui Jin Hospital, Shanghai Jiao Tong University School of Medicine, Shanghai 200025, China; 2 Institute of Cardiovascular Diseases, Shanghai Jiao Tong University School of Medicine, Shanghai 200025, China; 3 Yuhuangding Hospital, Qingdao University School of Medicine, Yantai 264000, China; 4 Department of Cardiology, Anqiu People’s Hospital, Weifang 261000, China

## Abstract

Whether serum calcium is associated with heart systolic function in patients with established coronary artery disease (CAD) and acute myocardial infarction (AMI) remains to be elucidated. This study is aimed to assess the association between serum calcium and left ventricular systolic dysfunction in a Chinese population of CAD. The cross-sectional study included 5938 CAD patients with and without AMI in China. The factors associated with AMI and left ventricular ejection fraction (LVEF) were evaluated. The data showed that AMI patients had lower serum calcium levels (2.11 ± 0.13 vs 2.20 ± 0.10 mmol/l, *P* < 0.001) than those without AMI. Multiple logistic regression analysis exhibited that serum calcium (OR: 0.000, 95% CI: 0.000–0.001) was one of the independent factors correlated with AMI. CAD patients with and without AMI when LVEF <50% had lower serum calcium levels than those when LVEF ≥50% respectively. Serum calcium was independently associated with LVEF and LVEF <50% in CAD patients with and without AMI respectively using multivariate analysis. The independent association between serum calcium and LVEF still existed among CAD patients when LVEF ≥50%. Serum calcium levels are significantly decreased following AMI. Low serum calcium is independently correlated with left ventricular systolic dysfunction in CAD patients with and without AMI.

Heart failure (HF) continues to be a major public health problem with high morbidity and mortality rates, despite the advances in medical treatment. Heart failure also has an enormous cost in terms of poor prognosis with an average mortality of about 30% in one year. Heart failure patients also have severe symptoms and a poor quality of life^1^,^2^. Heart failure can be classified as heart failure and reduced ejection fraction (HFREF), and heart failure and preserved ejection fraction (HFPEF). In combining individual patient data from 31 studies, the investigators showed that patients with HFREF had higher total mortality rates compared with those with HFPEF^3^. Myocardial ischemia and myocardial infarction (MI) are the important causes for reduced left ventricular ejection fraction (LVEF). Furthermore, left ventricular systolic dysfunction can significantly increase the mortalities among patients with coronary artery disease (CAD).

The central role of calcium in the sequence of myocardial excitation-contraction coupling and myocardial relaxation is well established. At the same time, previous studies suggested that hypocalcemic heart failure was found in patients with hypocalcemia-induced reversible cardiomyopathy and no underlying myocardial disease, and left ventricular systolic dysfunction could be improved after normalization of the serum calcium levels^4^,^5^,^6^,^7^. However, whether serum calcium levels are associated with heart systolic function in patients with established CAD and acute myocardial infarction (AMI) remains to be elucidated. Thus, we present this study aimed to assess the association between serum calcium and left ventricular systolic dysfunction in a Chinese population of CAD including AMI patients.

## Results

### Study population characteristics

Clinical characteristics of the 5938 CAD patients with and without AMI were shown respectively in Table 1 because the clinical characteristics and biochemical examination in patients with and without AMI were possibly quite different. The average age of all study subjects was 65.1 ± 10.4 years old. 4353 participants (73.3%) were man. The average LVEF was 63.0 ± 8.08% and prevalence of LVEF <50% in this population was 7.3%. In 4893 patients without AMI, 77.6% of them had chronic stable angina, and 22.4% had unstable angina. 63 subjects (2.3%) of angina patients had a history of impaired left ventricular systolic function. 98 subjects (4.3%) of angina patients had a history of heart failure, 2078 subjects (42.5%) had a history of percutaneous coronary intervention (PCI). The median troponin I values among angina patients were 0.01 ng/ml (range from 0.01 to 1.59 ng/ml). At the same time, there were still 1045 AMI patients. The median troponin I values among AMI patients were 19.80 ± 29.99 ng/ml (range from 0.01 to 103 ng/ml). A higher percentage of CAD patients with AMI were men, younger, and had current smoking compared with those without AMI (all *P* < 0.05). They also had lower LVEF, serum calcium, serum phosphate, plasma high-density lipoprotein cholesterol (HDL-C) levels, but higher plasma total cholesterol, low-density lipoprotein cholesterol (LDL-C), and fasting plasma glucose than those without AMI (all *P* < 0.001).

### Association between AMI and serum calcium

Serum calcium levels were lower in AMI patients. Furthermore, the independent association between AMI and serum calcium was assessed using multiple logistic regression analysis. The serum calcium levels entered the regression analysis as a linear variable. Other variables identified as statistically significant in the univariate analysis (with AMI vs without AMI) also entered the regression equation. Age and sex were also included as important demographic characteristics. Finally, lower serum calcium was one of the independent factors correlated with AMI with adjustment for other potential confounders including age, man, hypertension, current smoking, LVEF, serum phosphate, total cholesterol, LDL-C, HDL-C, and fasting plasma glucose (Table 2).

### Association between LVEF and serum calcium

Because of the strikingly different clinical characteristics including serum calcium between patients with and without AMI, association between LVEF and serum calcium was evaluated in patients with and without AMI respectively. Table 3 showed that a higher percentage of AMI patients with LVEF <50% were older, and had diabetes compared with those LVEF ≥50% (*P* < 0.001). They also had lower serum calcium, total cholesterol, total triglyceride, LDL-C levels, and estimated glomerular filtration rate (eGFR), but higher fasting plasma glucose than those with LVEF ≥50% (all *P* < 0.05). At the same time, another population of patients without AMI and with LVEF <50% also had lower serum calcium than those without AMI but LVEF ≥50% (*P* < 0.05).

In order to explore whether serum calcium was independently associated with LVEF or not, multiple linear regression analysis was used to evaluate the related factors with LVEF in patients with and without AMI respectively. Serum calcium, age, sex, and other variables identified as statistically significant in the univariate analysis (LVEF ≥50% vs LVEF <50%) entered the regression equation. The results in Table 4 showed that serum calcium (standard β coefficient: 0.109, *P* *<* 0.001) was independently associated with LVEF with adjustment for other potential confounders in AMI patients. At the same time, serum calcium (standard β coefficient: 0.055, *P* *<* 0.001) was also independently associated with LVEF in patients without AMI.

Furthermore, multiple logistic regression analysis was used to investigate the independent factors associated with LVEF <50%. The data in Table 5 exhibited that serum calcium (OR: 0.172, 95% CI: 0.034–0.872, *P* = 0.034, and OR: 0.674, 95%CI: 0.520–0.873, *P* = 0.003, respectively) was independently associated with LVEF <50% with adjustment for other potential confounders in patients with and without AMI respectively.

### Association between LVEF and serum calcium in patients with LVEF >50%

In fact, most of the participants enrolled in this study had LVEF values of more than 50%. We attempted to explore whether serum calcium continued to be independently associated with LVEF in patients with LVEF >50%. The data showed that serum calcium was still independently associated with LVEF with adjustment for other potential confounders in patients with higher LVEF regardless of the presence of AMI or not (Table 6).

## Discussion

The prevalence of and overall mortality from systolic heart failure has been increasing in recent decades. CAD and MI are important causes for heart systolic dysfunction. Mortalities are significantly increased in CAD and MI patients when left ventricular systolic dysfunction occurs^8^,^9^. Prevalence of LVEF <50% was 7.3% in the total population of this study and was even 12.3% among AMI patients. Thus, heart systolic dysfunction in CAD and AMI patients should receive more attention.

A higher percentage of AMI patients had current smoking habits, and had higher LDL-C, fasting plasma glucose, but lower HDL-C levels compared with those without AMI in this study. Of course, CAD patients with more cardiovascular risk factors tend to have increased opportunities to suffer from AMI. Serum calcium levels were significantly decreased when AMI occurred even if this trend was adjusted by other potential confounders. Although various studies also demonstrated that baseline higher serum calcium levels were found to be associated with the presence of calcified coronary atherosclerotic plaque and also an independent and prospective risk factor for new-onset of MI in patients with cardiovascular risk factors^10^,^11^. When the patients were suffering from AMI, serum calcium levels can be decreased for a periods of time. In fact, previous studies also reported that serum electrolytes including calcium, magnesium, and potassium can fall following AMI^12^,^13^. And furthermore, a cohort study suggested that low serum calcium was an independent predictor for in-hospital mortality in patients with acute ST-segment elevation myocardial infarction (STEMI). The authors of that study advised that serum calcium as a widely available serum biochemical index may be incorporated into the current established risk stratification model of STEMI patients^14^.

Whether the CAD patients suffered from AMI or not, serum calcium levels were significantly decreased if they had reduced LVEF in this study. Although a previous study reported that baseline higher serum calcium levels were independently associated with greater risk of incident HF in a population-based cohort^15^. That was possibly because high serum calcium tended to cause the new development of CAD and MI, then more opportunities of HF in the future. Nevertheless, low serum calcium levels can be present in established HF. In fact, several studies demonstrated that low serum calcium could be detected in heart failure patients^4^,^5^,^6^,^7^. However in our study, causal relationship between low serum calcium and heart systolic dysfunction remains to be elucidated and still need further study. Whether low calcium levels had affected the myocardial contractility in CAD patients in our study was unknown. Whether low serum calcium in patients with LVEF <50% was correlated with the use of medicine such as diuretics were also unclear. Whereas lower serum calcium was still accompanied by relatively lower LVEF even among patients with LVEF >50% in this study who had less opportunities receiving therapy of diuretics. Considering the roles of serum calcium in myocardial excitation-contraction coupling and cardiac electrophysiologic effect, we believe that low serum calcium may make sense in terms of disease severity or prognosis among patients with AMI or CAD patients with reduced heart systolic function.

Our study should also be interpreted within the context of its limitations. First, a relation between MI mortality and serum calcium had already been shown in a previous prospective cohort study. Our data just exhibited that low serum calcium was associated with the presence of AMI and low LVEF respectively. Second, subjects with left ventricular systolic dysfunction were not many and prevalence of LVEF <50% in the whole study group was only 7.3%, thus the analysis of independent link between low LVEF and low serum calcium might be impacted. Third, this study displayed that low serum calcium was associated with low LVEF in ischemic heart disease, but whether the association still existed or not in non-ischemic heart disease was unknown and need further investigations. Fourth, it should be cautioned that more coronary angiography was used in CAD diagnosis and risk assessment in clinical practice in China from this study. In fact, more functional tests including treadmill exercise test, exercise or pharmacological stress with nuclear myocardial perfusion imaging or echocardiography should be recommended in clinical practice. Finally, a casual link between serum calcium levels and LVEF, and related mechanism had not been illuminated in this study and need further studies in the future.

In summary, serum calcium levels are significantly decreased following AMI. Serum calcium levels are lower with decreased LVEF. Low serum calcium is independently correlated with left ventricular systolic dysfunction in CAD patients with and without AMI.

## Methods

### Study population

This study was cross-sectional and focused on CAD patients in China. At first, totally 6693 participants aged over 25 years old and consecutively hospitalized in department of cardiology of Shanghai Rui Jin hospital from January 2011 to December 2014, were enrolled in the study. The participants were hospitalized to ascertain the diagnosis among suspected CAD or AMI candidates, or perform risk stratification and receive further intervention among established CAD or AMI patients. All the participants in this study received coronary angiography (CAG). The main indication for CAG among subjects without AMI were as follows: (1) When subjects had chest discomfort, impaired left ventricular systolic function, or heart failure, and had a noninvasive test suggesting uncertain myocardial ischemia as well, they received CAG to ascertain the diagnosis of CAD; (2) When subjects with established CAD including PCI history, old myocardial infarction, or significant coronary stenosis confirmed by coronary CT angiography still had chest comfort after medical therapy, they further received CAG for risk assessment and evaluating whether coronary revascularization was needed. CAD was diagnosed by CAG when diameter stenosis was >50% in at least one main coronary artery, or the presence of AMI, or a history of confirmed MI, or a history of revascularization by PCI, or coronary artery bypass graft (CABG). The diagnosis of AMI was confirmed according to the third universal definition of myocardial infarction in 2012, in which an increase in the cardiac biological markers (preferably cardiac troponin) with at least one value higher than the 99% of the reference level and having at least one of the following signs. These signs included symptoms of acute ischemia, new or accepted new ST-segment-T wave changes or newly formed left bundle branch block, pathological Q waves on ECG, newly occurring tissue loss or regional wall motion dysfunction in living myocardium, and thrombosis within the coronary artery on angiography^16^. Exclusion criteria included congenital heart disease, valvular heart disease, hyperthyroidism, hyperparathyroidism, hypoparathyroidism, acute infected diseases, acute pancreatitis, liver failure, pregnancy, mental disorder, or cancer. After the subjects with exclusion criteria or incomplete data were removed, 5938 participants with diagnosis of CAD including angina or AMI entered the statistical analysis.

The study complied with the Declaration of Helsinki. It was also approved by the ethics committee of Shanghai Jiao Tong University and informed consent was obtained from all the participants prior to enrollment.

### Blood sampling and laboratory analyzes

The blood samples were collected from each patient after admission. The concentrations of serum calcium (mmol/l) and serum phosphate (mmol/l) were measured by the automatic biochemical analyzer (Beckman Coulter, CA, USA). Cardiac troponin I (ng/ml) was analyzed by an immunochemiluminometric assay (Access AccuTnI, Beckman Coulter, CA, USA). Meanwhile, the levels of plasma total cholesterol (mmol/l), total triglyceride (mmol/l), HDL-C (mmol/l), LDL-C (mmol/l), plasma glucose (mmol/l), and serum creatinine (umol/l) were analyzed by the automatic biochemical analyzer. Laboratory test results were generated by personnel blinded to the clinical characteristics of the study participants.

### Echocardiographic measurements

Two echocardiographers blinded to the biochemical examination results of the study participants performed all echocardiographic measurements using the Phillips IE33 device according to the American Society of Echocardiography (ASE) recommendations. M-mode, two-dimensional, and colour Doppler images were first recorded, and then analyzed offline. LVEF assessment was based on two-dimensional echocardiography using the quantitative two-dimensional biplane volumetric Simpson method from 4- and 2-chamber views. Inter- or intra-observer reproducibility was assessed among 25 randomly selected patients. No significant difference was found (inter-observer: mean difference: 2.0 ± 0.21%, *P* = 0.431; intra-observer: mean difference: 1.2 ± 0.17%, *P* = 0.698).

### Clinical data collection

A case report form was developed to record the general characteristics, clinical diagnosis, medical history, and biochemical examination. eGFR was calculated using serum creatinine according to the CKD-EPI China equation with adjusted coefficient of 1.1 for the Chinese population^17^. Current smoking was determined when subjects were smoking currently and more than one cigarette daily in at least one year continuously. Hypertension was diagnosed when systolic blood pressure (SBP) ≥140 mmHg, or diastolic blood pressure (DBP) ≥90 mmHg, or being actively treated with anti-hypertension drugs. Diabetes mellitus was diagnosed by a fasting plasma glucose test showing ≥7.0 mmol/l, or by a random plasma glucose test showing ≥11.1 mmol/l, or when they were actively receiving therapy using insulin or oral medications for diabetes.

### Statistical analysis

The final data were analyzed using the software program SPSS 13.0 (SPSS Inc., Chicago, IL, USA). The continuous variables with normal distribution were expressed as the mean  ±  standard deviation, whereas continuous variables with a skewed distribution were reported as the median (interquartile range). Categorical variables were expressed as frequency and percentage. The chi-square test was used to compare categorical variables between several groups. The independent-sample t-test or Mann-Whitney U test were used to compare continuous variables with normal or skewed distribution between two groups respectively. Multiple logistic regression analysis which included variables identified as statistically significant in the univariate analysis, was used to assess the independent association between AMI or LVEF <50% and related factors. The odds ratio (OR) and 95% confidence interval (95% CI) were calculated. Multiple linear regression analysis was used to assess the independent association between LVEF and related factors. Standard β coefficient, β coefficient, and β coefficient’s 95% confidence interval were determined. P < 0.05, which is two-sided, was considered significant.

## Additional Information

**How to cite this article**: Wang, Y. *et al.* Low serum calcium is associated with left ventricular systolic dysfunction in a Chinese population with coronary artery disease. *Sci. Rep.* **6**, 22283; doi: 10.1038/srep22283 (2016).

## Figures and Tables

**Table 1:** Clinical characteristics of coronary artery disease patients with and without AMI.

Variables	All(n = 5938)	Without AMI(n = 4893)	With AMI(n = 1045)	*P*value
Age, yrs	65.1 ± 10.4	65.2 ± 10.1	64.4 ± 11.9	0.019
Men (n,%)	4352(73.3%)	3522(72.0%)	830(79.4%)	<0.001
BMI (kg/m^2^)	24.7 ± 1.40	24.7 ± 1.41	24.8 ± 1.48	0.371
Current smoking (n,%)	1346(22.7%)	1000(20.4%)	346(33.1%)	<0.001
Diabetes mellitus (n,%)	2099(35.3%)	1733(35.4%)	366(35.0%)	0.809
Hypertension (n,%)	3972(66.9%)	3326(68.0%)	646(61.8%)	<0.001
History of PCI (n,%)	2291(38.6%)	2078(42.5%)	213(20.4%)	<0.001
Current PCI procedure (n,%)	4251(71.6%)	3254(66.5%)	997(95.4%)	<0.001
SBP (mmHg)	135 ± 21.0	135 ± 21.2	134 ± 20.3	0.126
DBP (mmHg)	75.5 ± 11.8	75.5 ± 11.9	75.3 ± 11.5	0.637
LVEF (%)	63.0 ± 8.08	63.8 ± 7.83	59.0 ± 8.06	<0.001
LVEF <50% (n,%)	431(7.3%)	302(6.2%)	129(12.3%)	<0.001
Cardiac troponin I (ng/ml)	0.01(0.01–0.04)	0.01(0.01–0.01)	5.07(1.16–22.7)	<0.001
Serum creatinine (umol/l)	78(67–91)	78(67–91)	79(69–92)	0.070
eGFR (ml/min/1.73 m^2^)	89.9 ± 20.3	89.8 ± 20.0	90.5 ± 21.8	0.291
Serum calcium (mmol/l)	2.19 ± 0.11	2.20 ± 0.10	2.11 ± 0.13	<0.001
Serum phosphate (mmol/l)	1.15 ± 0.23	1.16 ± 0.21	1.10 ± 0.31	<0.001
Total cholesterol (mmol/l)	4.01 ± 1.07	3.95 ± 1.06	4.33 ± 1.07	<0.001
Total triglyceride (mmol/l)	1.73 ± 1.18	1.74 ± 1.19	1.70 ± 1.14	0.450
LDL-C (mmol/l)	2.38 ± 0.87	2.32 ± 0.86	2.67 ± 0.86	<0.001
HDL-C (mmol/l)	1.07 ± 0.28	1.09 ± 0.28	1.03 ± 0.27	<0.001
Fasting plasma glucose (mmol/l)	5.64 ± 1.85	5.51 ± 1.66	6.26 ± 2.47	<0.001

AMI: acute myocardial infarction; BMI: body mass index; SBP, systolic blood pressure; DBP, diastolic blood pressure; LVEF: left ventricular ejection fraction; LDL-C: low-density lipoprotein cholesterol; HDL-C: high-density lipoprotein cholesterol; eGFR: estimated glomerular filtration rate. Values are means  ±  SD, medians (interquartile range), or numbers with percentage in parenthesis.

**Table 2:** Factors associated with AMI using multiple logistic regression analysis.

Variables	OR	95% confidence interval	*P*value
Age (years)	0.988	0.979–0.996	0.003
Man (1 = yes, 0 = no)	1.053	0.849–1.308	0.637
Hypertension (1 = yes, 0 = no)	0.925	0.783–1.093	0.359
Current smoking (1 = yes, 0 = no)	1.683	1.398–2.025	<0.001
LVEF (%)	0.950	0.941–0.958	<0.001
Serum calcium (mmol/l)	0.000	0.000–0.001	<0.001
Serum phosphate (mmol/l)	0.430	0.301–0.614	<0.001
Total cholesterol (mmol/l)	1.346	1.095–1.654	0.005
LDL-C (mmol/l)	1.182	0.925–1.511	0.182
HDL-C (mmol/l)	0.592	0.423–0.827	0.002
Fasting plasma glucose (mmol/l)	1.148	1.105–1.192	<0.001

AMI: acute myocardial infarction; LVEF: left ventricular ejection fraction; LDL-C: low-density lipoprotein cholesterol; HDL-C: high-density lipoprotein cholesterol. The odds ratio (OR) of factors associated with AMI was calculated using multiple logistic regression analysis.

**Table 3:** Clinical characteristics of coronary artery disease patients with EF <50% or ≥50%.

Variables	Without AMI (n = 4893)		*P*value	With AMI (n = 1045)		*P*value
—	LVEF ≥50% (n = 4591)	LVEF <50% (n = 302)	—	LVEF ≥50% (n = 916)	LVEF <50% (n = 129)	—
Age, yrs	65.2 ± 10.1	66.2 ± 10.5	0.099	63.6 ± 11.6	69.7 ± 12.3	<0.001
Men (n,%)	3274(71.3%)	248(82.1%)	<0.001	733(80.0%)	97(75.2%)	0.204
BMI (kg/m^2^)	24.7 ± 1.41	24.7 ± 1.43	0.823	24.8 ± 1.47	24.6 ± 1.53	0.294
Current smoking (n,%)	937(20.4%)	63(20.9%)	0.851	312(34.1%)	34(26.4%)	0.082
Diabetes mellitus (n,%)	1596(34.8%)	137(45.4%)	<0.001	301(32.9%)	65(50.4%)	<0.001
Hypertension (n,%)	3138(68.4%)	188(62.3%)	0.028	565(61.7%)	81(62.8%)	0.808
SBP (mmHg)	135 ± 21.3	133 ± 19.4	0.130	134 ± 20.1	131 ± 21.4	0.111
DBP (mmHg)	75.5 ± 11.9	75.4 ± 12.1	0.887	75.5 ± 11.3	74.1 ± 12.6	0.206
LVEF (%)	65.2 ± 5.36	41.7 ± 5.93	<0.001	61.2 ± 5.91	43.8 ± 5.20	<0.001
Cardiac troponin I (ng/ml)	0.01(0.01–0.01)	0.02(0.01–0.04)	<0.001	4.99(1.13–20.9)	6.66(1.29–38.0)	0.066
Serum creatinine (umol/l)	78(67–90)	86(71–97)	<0.001	79(68–90)	89(72–103)	<0.001
eGFR (ml/min/1.73 m^2^)	90.2 ± 19.6	83.7 ± 24.2	<0.001	92.2 ± 20.9	78.9 ± 24.3	<0.001
Serum calcium (mmol/l)	2.20 ± 0.10	2.19 ± 0.11	0.024	2.11 ± 0.12	2.07 ± 0.12	<0.001
Serum phosphate (mmol/l)	1.16 ± 0.20	1.16 ± 0.23	0.947	1.09 ± 0.30	1.11 ± 0.34	0.647
Total cholesterol (mmol/l)	3.94 ± 1.05	3.98 ± 1.22	0.604	4.35 ± 1.08	4.14 ± 0.96	0.038
Total triglyceride (mmol/l)	1.74 ± 1.20	1.60 ± 1.02	0.040	1.74 ± 1.16	1.48 ± 0.89	0.022
LDL-C (mmol/l)	2.31 ± 0.85	2.39 ± 0.99	0.146	2.69 ± 0.87	2.50 ± 0.80	0.026
HDL-C (mmol/l)	1.09 ± 0.28	1.04 ± 0.27	0.005	1.02 ± 0.26	1.06 ± 0.30	0.170
Fasting plasma glucose (mmol/l)	5.49 ± 1.63	5.82 ± 2.12	0.001	6.18 ± 2.38	6.87 ± 2.94	0.004

AMI: acute myocardial infarction; LVEF: left ventricular ejection fraction; BMI: body mass index; SBP, systolic blood pressure; DBP, diastolic blood pressure; LDL-C: low-density lipoprotein cholesterol; HDL-C: high-density lipoprotein cholesterol; eGFR: estimated glomerular filtration rate. Values are means  ±  SD, medians (interquartile range), or numbers with percentage in parenthesis.

**Table 4:** Factors associated with LVEF using multiple linear regression analysis.

Variables	Standard β coefficient	β coefficient	β coefficient’s 95% confidence interval	*P*value
Without AMI	—	—	—	—
Age (years)	0.052	0.040	0.012 to 0.068	0.005
Man (1 = yes, 0 = no)	−0.108	−1.876	−2.394 to −1.358	<0.001
Diabetes mellitus (1 = yes, 0 = no)	−0.053	−0.859	−1.371 to −0.347	0.001
Hypertension (1 = yes, 0 = no)	0.039	0.651	0.176 to 1.126	0.007
eGFR (ml/min/1.73 m^2^)	0.156	0.061	0.047 to 0.074	<0.001
Serum calcium (mmol/l)	0.055	4.143	1.923 to 6.362	<0.001
Fasting plasma glucose (mmol/l)	−0.044	−0.207	−0.355 to −0.059	0.006
HDL-C (mmol/l)	0.029	0.817	−0.070 to 1.705	0.071
Total triglyceride (mmol/l)	0.039	0.257	0.061 to 0.453	0.010
Cardiac troponin I (ng/ml)	−0.113	−11.662	−14.554 to −8.771	<0.001
With AMI	—	—	—	—
Age (years)	−0.104	−0.069	−0.122 to −0.016	0.011
Man (1 = yes, 0 = no)	−0.073	−1.437	−2.410 to −1.368	0.029
Diabetes mellitus (1 = yes, 0 = no)	−0.020	−0.325	−1.471 to 0.821	0.578
eGFR (ml/min/1.73 m^2^)	0.148	0.053	0.026 to 0.081	<0.001
Serum calcium (mmol/l)	0.109	6.740	2.846 to 10.633	0.001
Fasting plasma glucose (mmol/l)	−0.125	−0.398	−0.624 to −0.173	0.001
Total triglyceride (mmol/l)	0.045	0.330	−0.252 to 0.870	0.280
Total cholesterol (mmol/l)	0.045	0.330	−1.179 to 1.838	0.668
LDL-C (mmol/l)	−0.089	−0.811	−2.599 to 0.978	0.374

LVEF: left ventricular ejection fraction; AMI: acute myocardial infarction; HDL-C: high-density lipoprotein cholesterol; LDL-C: low-density lipoprotein cholesterol; eGFR: estimated glomerular filtration rate.

**Table 5:** Factors associated with LVEF <50% using multiple logistic regression analysis.

Variables	OR (95% CI)	*P*value	Variables	OR (95% CI)	*P*value
Without AMI			With AMI		
Age (years)	0.992 (0.977–1.007)	0.315	Age (years)	1.025 (1.002–1.049)	0.035
Man (1 = yes, 0 = no)	1.772 (1.276–2.462)	0.001	Man (1 = yes, 0 = no)	1.082 (0.641–1.828)	0.767
Diabetes mellitus (1 = yes, 0 = no)	1.497 (1.136–1.972)	0.004	Diabetes mellitus (1 = yes, 0 = no)	2.064 (1.307–3.261)	0.002
Hypertension (1 = yes, 0 = no)	0.674 (0.520–0.873)	0.003	Serum calcium (mmol/l)	0.172 (0.034–0.872)	0.034
eGFR (ml/min/1.73 m^2^)	0.980 (0.974–0.987)	<0.001	Fasting plasma glucose (mmol/l)	1.044 (0.963–1.132)	0.298
Serum calcium (mmol/l)	0.213 (0.061–0.741)	0.015	eGFR (ml/min/1.73 m^2^)	0.985 (0.974–0.995)	0.003
Fasting plasma glucose (mmol/l)	1.079 (1.010–1.153)	0.024	Total triglyceride (mmol/l)	0.832 (0.623–1.111)	0.213
HDL-C (mmol/l)	0.653 (0.387–1.104)	0.112	Total cholesterol (mmol/l)	1.309 (0.723–2.368)	0.374
Total triglyceride (mmol/l)	0.838 (0.726–0.968)	0.016	LDL-C (mmol/l)	0.615 (0.301–1.257)	0.182
Cardiac troponin I (ng/ml)	13.263(5.076–34.653)	<0.001	—	—	—

LVEF: left ventricular ejection fraction; AMI: acute myocardial infarction; HDL-C: high-density lipoprotein cholesterol; LDL-C: low-density lipoprotein cholesterol; eGFR: estimated glomerular filtration rate. The odds ratio (OR) with 95% confidence interval (95% CI) of various factors associated with LVEF <50% was produced using multiple logistic regression analysis.

**Table 6:** Factors associated with LVEF in subjects with LVEF ≥50% using multiple linear regression analysis.

Variables	Standard β coefficient	β coefficient	β coefficient’s 95% confidence interval	*P*value
Without AMI	—	—	—	—
Age (years)	0.035	0.019	−0.001 to 0.039	0.069
Man (1 = yes, 0 = no)	−0.106	−1.257	−1.627 to −0.886	<0.001
Diabetes mellitus(1 = yes, 0 = no)	−0.045	−0.498	−0.867 to −0.129	0.008
Hypertension (1 = yes, 0 = no)	0.009	0.098	−0.244 to 0.440	0.574
eGFR (ml/min/1.73 m^2^)	0.112	0.030	0.020 to 0.040	<0.001
Serum calcium (mmol/l)	0.043	2.282	0.676 to 3.888	0.005
Fasting plasma glucose (mmol/l)	−0.014	−0.045	−0.154 to 0.063	0.416
HDL-C (mmol/l)	0.024	0.462	−0.177 to 1.101	0.156
Total triglyceride (mmol/l)	0.015	0.066	−0.074 to 0.206	0.354
Cardiac troponin I (ng/ml)	−0.054	−4.157	−6.390 to −1.924	<0.001
With AMI	—	—	—	—
Age (years)	−0.073	−0.037	−0.080 to 0.006	0.093
Man (1 = yes, 0 = no)	−0.099	−1.488	−2.547 to −0.428	0.006
Diabetes mellitus (1 = yes, 0 = no)	0.057	0.718	−0.245 to 1.681	0.144
eGFR (ml/min/1.73 m^2^)	0.082	0.023	0.001 to 0.045	0.045
Serum calcium (mmol/l)	0.082	3.837	0.627 to 7.048	0.019
Fasting plasma glucose (mmol/l)	−0.134	−0.332	−0.525 to −0.140	0.001
Total triglyceride (mmol/l)	0.037	0.190	−0.257 to 0.637	0.404
Total cholesterol (mmol/l)	0.073	0.399	−0.815 to 1.614	0.519
LDL-C (mmol/l)	−0.180	−1.228	−2.669 to 0.213	0.095

LVEF: left ventricular ejection fraction; AMI: acute myocardial infarction; HDL-C: high-density lipoprotein cholesterol; LDL-C: low-density lipoprotein cholesterol; eGFR: estimated glomerular filtration rate.

## References

[b1] Azad N. & Lemay G. Management of chronic heart failure in the older population. J Geriatr Cardiol. 11, 329–337 (2014). 25593582 10.11909/j.issn.1671-5411.2014.04.008 PMC4292097

[b2] Jeevanantham V., Daubert J. P. & Zareba W. Cardiac resynchronization therapy in heart failure patients: an update. Cardiol J. 16, 197–209 (2009). 19437393

[b3] Meta-analysis Global Group in Chronic Heart Failure (MAGGIC). The survival of patients with heart failure with preserved or reduced left ventricular ejection fraction: an individual patient data meta analysis. Eur Heart J. 33, 1750–1757 (2012). 21821849 10.1093/eurheartj/ehr254

[b4] Newman D. B. *et al.* Reversible cardiac dysfunction associated with hypocalcemia: a systematic review and meta-analysis of individual patient data. Heart Fail Rev. 19, 199–205 (2014). 23355181 10.1007/s10741-013-9371-1

[b5] Suzuki T., Ikeda U., Fujikawa H., Saito K. & Shimada K. Hypocalcemic heart failure: a reversible form of heart muscle disease. Clin Cardiol. 21, 227–228 (1998). 9541771 10.1002/clc.4960210319 PMC6656229

[b6] Behaghel A. & Donal E. Hypocalcaemia-induced transient dilated cardiomyopathy in elderly: a case report. Eur J Echocardiogr. 12, E38 (2011). 21846649 10.1093/ejechocard/jer105

[b7] Catalano A., Basile G. & Lasco A. Hypocalcemia: a sometimes overlooked cause of heart failure in the elderly. Aging Clin Exp Res. 24, 400–403 (2012). 23238316 10.1007/BF03325272

[b8] Januzzi J. L. Jr., Filippatos G., Nieminen M. & Gheorghiade M. Troponin elevation in patients with heart failure: on behalf of the third Universal Definition of Myocardial Infarction Global Task Force: Heart Failure Section. Eur Heart J. 33, 2265–2271 (2012). 22745356 10.1093/eurheartj/ehs191

[b9] Weir R. A. & McMurray J. J. Epidemiology of heart failure and left ventricular dysfunction after acute myocardial infarction. Curr Heart Fail Rep. 3, 175–180 (2006). 17129511 10.1007/s11897-006-0019-5

[b10] Lind L., Skarfors E., Berglund L., Lithell H. & Ljunghall S. Serum calcium: a new, independent, prospective risk factor for myocardial infarction in middle-aged men followed for 18 years. J Clin Epidemiol. 50, 967–973 (1997). 9291883 10.1016/s0895-4356(97)00104-2

[b11] Shin S. *et al.* Impact of serum calcium and phosphate on coronary atherosclerosis detected by cardiac computed tomography. Eur Heart J. 33, 2873–2881 (2012). 22719023 10.1093/eurheartj/ehs152

[b12] Abraham A. S., Rosenman D., Meshulam Z., Zion M. & Eylath U. Serum, lymphocyte, and erythrocyte potassium, magnesium, and calcium concentrations and their relation to tachyarrhythmias in patients with acute myocardial infarction. Am J Med. 81, 983–988 (1986). 3799659 10.1016/0002-9343(86)90392-x

[b13] Ising H. *et al.* Alterations of electrolytes in serum and erythrocytes after myocardial infarction. Magnesium. 6, 192–200 (1987). 3669728

[b14] Lu X. *et al.* Association of admission serum calcium levels and in-hospital mortality in patients with acute ST-elevated myocardial infarction: an eight-year, single-center study in China. PLoS One. 9, e99895 (2014). 24926660 10.1371/journal.pone.0099895 PMC4057419

[b15] Lutsey P. L. *et al.* Serum magnesium, phosphorus, and calcium are associated with risk of incident heart failure: the Atherosclerosis Risk in Communities (ARIC) Study. Am J Clin Nutr. 100, 756–764 (2014). 25030784 10.3945/ajcn.114.085167 PMC4135486

[b16] Thygesen K. *et al.* Third universal definition of myocardial infarction. Circulation. 126, 2020–2035 (2012). 22923432 10.1161/CIR.0b013e31826e1058

[b17] Teo B. W. *et al.* GFR estimating equations in a multiethnic Asian population. Am J Kidney Dis. 58, 56–63 (2011). 21601325 10.1053/j.ajkd.2011.02.393

